# Plasma Plume Oscillations Monitoring during Laser Welding of Stainless Steel by Discrete Wavelet Transform Application

**DOI:** 10.3390/s100403549

**Published:** 2010-04-08

**Authors:** Teresa Sibillano, Antonio Ancona, Domenico Rizzi, Valentina Lupo, Luigi Tricarico, Pietro Mario Lugarà

**Affiliations:** 1 CNR-INFM Regional Laboratory “LIT^3^”, via Amendola 173, I-70126 Bari, Italy; E-Mails: ancona@fisica.uniba.it (A.A.); rizzi@fisica.uniba.it (D.R.); 2 Dipartimento Interateneo di Fisica “M. Merlin”, Università and Politecnico of Bari, via Amendola 173, I-70126 Bari, Italy; E-Mail: lugara@fisica.uniba.it (P.M.L.); 3 Dipartimento di Ing. Meccanica e Gestionale, Politecnico di Bari, I-70126 Bari, Italy; E-Mail: tricaric@fisica.uniba.it (L.T.)

**Keywords:** optical sensor, laser welding, plasma spectroscopy

## Abstract

The plasma optical radiation emitted during CO_2_ laser welding of stainless steel samples has been detected with a Si-PIN photodiode and analyzed under different process conditions. The discrete wavelet transform (DWT) has been used to decompose the optical signal into various discrete series of sequences over different frequency bands. The results show that changes of the process settings may yield different signal features in the range of frequencies between 200 Hz and 30 kHz. Potential applications of this method to monitor in real time the laser welding processes are also discussed.

## 1. Introduction

Optical sensors for laser welding process monitoring are based on the detection of the plasma plume emission, the thermal radiation of the weld pool and the reflected laser light [1,2]. Among the approaches for the development of optical sensors, the most effective are the systems based on the measurement of the spatially integrated optical intensity or the spectroscopic analysis of the VIS/UV emission from the plasma plume [3–5].

Spectroscopic analysis of the plasma plume optical emission is a valuable approach for monitoring the quality of the process, since it gives information about the plasma plume features and therefore about the keyhole dynamics. Furthermore, the spectroscopic approach is non intrusive, low cost and easy to embed in automated systems [1–9].

Monitoring the light intensity from the plasma produced during laser welding is a common diagnostic method and yields information on the presence of defects and on the depth of penetration. A number of quality monitoring systems based on one or more photodiodes have been reported by several authors [10–12].

In spite of the extensive literature on the spectroscopic characterization of the plasma plume [1–9], and the light intensity analysis [10–12], only few studies have so far focused on the description of its oscillations arising from the instabilities of the process.

During laser welding process the keyhole is kept open by a pressure equilibrium. The pressure balance is continuously influenced by the process parameters, leading to oscillations of the keyhole and the melt pool. The fluctuations influence the expanding plasma dynamics and its oscillations [13–17].

Assuming that the fluctuations of the plasma plume are directly associated with the melt pool and keyhole instabilities, we have examined the oscillations of the optical signal detected by a Si-PIN photodiode collecting the broadband optical radiation generated during laser welding of AISI304 stainless steel plates in butt-joint configuration. Frequency analyses of the optical signals have been carried out by using the Discrete Wavelet Transform (DWT) tool.

The wavelet time-frequency analysis method, whose main features will be discussed in the following subsection, has been used in previous works to study signals of diverse nature (optical, airborne acoustic or electric) generated during laser welding [12,18,19], friction stir welding [20] and resistance spot welding processes [21]. This technique was realized to be a tool for recognizing transitions of welding states and identifying defects. Nonetheless, in case of acoustic and electric sensors the noisy environment of industrial welding workshops generally prevents their employment at a production scale.

In case of photodiode-based sensors a wavelet analysis of the optical emission of laser welding processes has been already attempted [12] in the frequency range below 5 kHz, typical of the keyhole radial oscillations as predicted by the models [13–17]. Fluctuations of the frequency components of the signal have been observed in case of poor penetration, corresponding to the points where the photodiode signal has a break [12].

In this paper, we have analyzed the optical signal in the time-frequency domain in the range up to 30 kHz, where the axial-azimuthal oscillations of the keyhole, mostly related to the weld penetration, are predicted.

The objective is to explore the potentiality of the DWT signal analysis technique to indicate defective parts of the joints when it is not possible to clearly distinguish them from a mere qualitative analysis of the acquired photodiode signal. Two defect causes have been investigated: lack of incident laser power and ineffective inert gas shielding.

### 1.1. Discrete Wavelet Analysis

The Fourier analysis is one of the most valuable and frequently used tools in signal processing and analysis. This tool decomposes a signal into sinusoidal constituents of several frequencies, transforming a signal in the time domain into its counterpart in the frequency domain. Fourier methods are not always a good solution to analyse the signal, especially for signals that undergo sudden changes, fluctuations or discontinuities.

In some cases, wavelet analysis is very effective because it provides a simple approach for dealing with local aspects of a signal. Actually, wavelet transform is capable of providing the time and frequency information simultaneously, hence giving a time-frequency representation of the signal. Unlike the Fourier transform that gives the precise frequency information, the wavelet transform provides band frequency information in the time domain. For this reason wavelet analysis is a promising tool for process monitoring due to its capability of time-frequency representations.

There are two different kinds of wavelet transform (WT): continuous and discrete [22,23]. Continuous wavelet analysis allows a general function of time to be decomposed into a series of basic functions, called wavelets, of different lengths and different positions along the time axis. A particular feature of the signal can be located from the positions of the wavelet into which it is decomposed. This allows the changing spectral composition of non-stationary signals to be measured and compared.

The wavelet transform employed in this work is the discrete one, where filters of different cut-off frequencies are used to analyze the signal at different scales.

The procedure starts with passing the signal x[n] through a digital low-pass filter with impulse response g[n] and a high-pass filter h[n]. The output of the low-pass filter is called approximation whilst the output of the high-pass filter is called detail. Filtering a signal corresponds to the mathematical operation of convolution of the signal with the impulse response of the filter. The convolution operation in discrete time is defined as follows:

$$\begin{array}{l} y_{\text{low}}[n]=x[n]*g[n]=\sum_{k=-\infty }^{\infty }x[k]\;\cdot \;g[2n-k]\\ y_{\text{high}}[n]=x[n]*h[n]=\sum_{k=-\infty }^{\infty }x[k]\;\cdot \;h[2n-k] \end{array}$$

**Figure 1.**
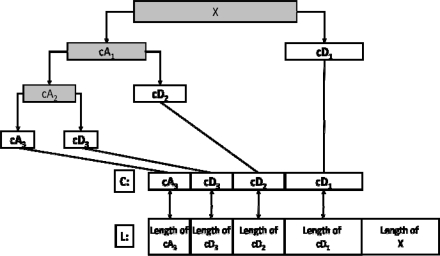
Level-3 decomposition example. A_1_, A_2_,…, A_n_ represent the approximation coefficients and D_1_, D_2_, .., D_n_ the details coefficients.

This decomposition has halved the time resolution since only half of each filter output characterises the signal. However, each output has halved the frequency band of the input so the frequency resolution has been doubled. This procedure can be repeated for further decomposition, each time decomposing successive approximations, and one signal is broken down into many components. In this way, one can get wavelet decomposition tree, shown in Figure 1.

Due to the decomposition process the input signal must be a multiple of 2n where n is the number of levels. Depending on the signal nature as well as the frequency band of interest, one can select suitable number of levels. By its structure, the whole range is divided into n frequency ranges, where n is the number of suitable levels. The wavelet decomposition returns the decomposition of the signal at level n. The output decomposition structure contains the wavelet decomposition vector C, that indicates the intensity of the selected level, and the length of this vector L, which depends on the chosen level n.

In this preliminary study, we examine how the ranges of frequency are influenced by the welding conditions and the occurrence of defects.

## 2. Experimental Set-Up

The welding tests were carried out by using a CO_2_ laser with maximum output power of 2.5 kW in continuous wave regime. The laser beam is focused onto the workpiece by a 200 mm focal length water-cooled parabolic mirror. The laser source is coupled to a fully-automated robotic cell (Ravasi LC 1,000). The plasma optical emission was collected by a quartz collimator of 6 mm focal length.

The collected light was transmitted to a Si-PIN photodiode (320−1,000 nm spectral sensitivity) by an 200 μm core-diameter optical fiber. The signals were pre-amplified and fed to a data acquisition processor. The sampling time was 16 μs, corresponding to a maximum frequency of 30 kHz.

The welding trials were performed on 2 mm-thick plates of AISI304 stainless steel in a butt-joint configuration.

The whole frequency range of the signal acquired from the photodiode was divided into seven levels of decomposition. The observed bandwidths are reported in Table 1. We examined how the ranges of frequency are influenced by the welding conditions and the occurrence of defects. For this purpose, the results obtained by the wavelet decomposition have been depicted in bi-dimensional colour maps that illustrate the absolute value of decomposition vector C distribution, for each level, from zero to its maximum value. This approach allows to clearly distinguish the frequency range most highly correlated to the process conditions.

## 3. Results and Discussion

A series of welding tests have been preliminarily performed aiming to find the most suitable process settings to reliably produce sound butt-joints. The optimized welding parameters using Helium as shielding gas are: laser power P = 1.8 kW and v = 80 mm/s.

**Figure 2.**
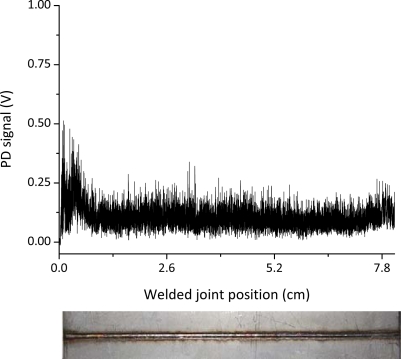
(a) Photodiode signal corresponding to a defect-free weld (P = 1.8 kW, v = 80 mm/s, Q_He_ = 100 Nl/min); (b) Top view of the welded joint.

Figure 2 reports the typical photodiode signal acquired during a laser welding process carried out under optimal welding conditions. Apart from some small instabilities at the ignition of the process, the signal stays almost constant during all the weld.

**Figure 3.**
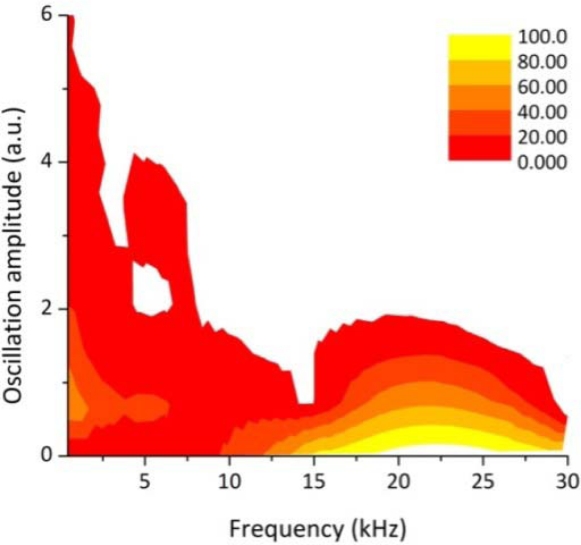
Relative amplitude of the oscillation frequency bands of the optical radiation emitted by the plasma plume during a welding process performed under optimal conditions (the color scale indicates the distribution in % for each frequency band).

The map in Figure 3 illustrates the distribution of the relative oscillation intensities in the frequency range between 200 Hz and 30 kHz, calculated by applying the Discrete Wavelet Transform to the signal reported in Figure 2. It can be noticed that in the range between 200 Hz and 10 kHz the oscillations amplitudes are significantly more intense than at higher frequency bands. More than 90% of the frequency components in the range between 15 and 30 kHz have an amplitude close to zero. Therefore, it can be argued that in stable and sound welding conditions the dominant frequency band of the plasma optical emission oscillations lies in the range between 200 Hz and 15 kHz.

**Figure 4.**
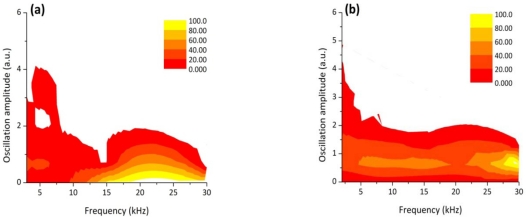

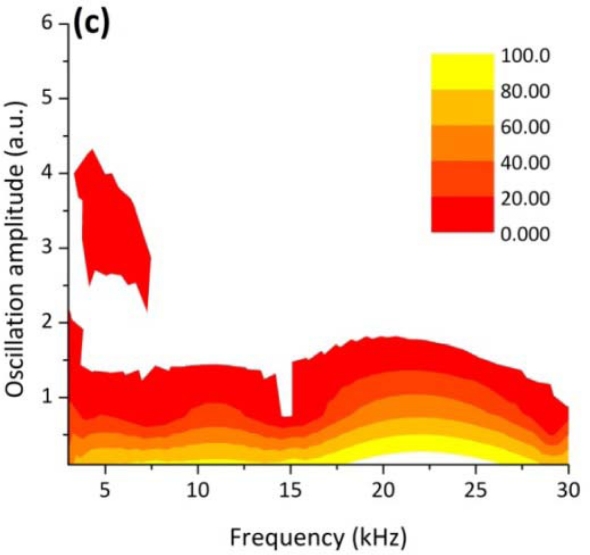
DWT oscillation frequency maps calculated for each joint segment (the color scale indicates the distribution in % for each frequency band): zone (a), (b) and (c) indicate three sectors of the welding

The photodiode signal depicted in Figure 2 was then divided into three zones and the DWT was calculated for each segment in order to see if any difference could be noticed on the frequency distributions thus checking the reliability of this technique. In Figure 4 the relative oscillation amplitudes as a function of the frequency bands are reported corresponding to the three different weld segments. The frequency distributions are nearly equivalent among the three graphs.

The capability of the wavelet analysis to detect variations of the welding conditions induced by changing of the main process parameters was further examined by decreasing the laser power from 1.8 kW to 0.9 kW during the run and keeping the welding speed at a constant value of 80 mm/s.

**Figure 5.**
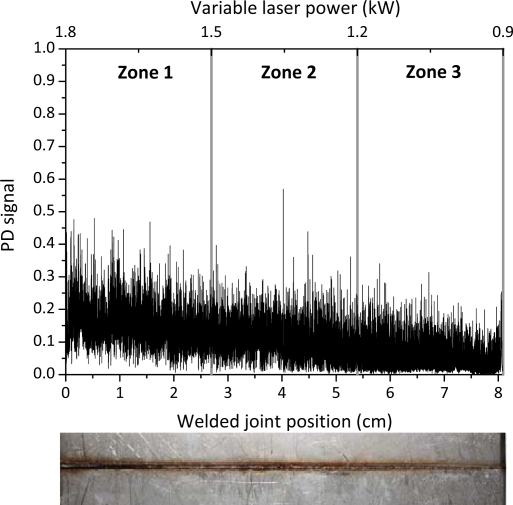
(a) Photodiode signal detected during a weld in which the laser power was varied from P = 1.8 kW to P = 0.9 kW; (b) Top view of the welded joint.

The acquired optical signal as a function of the welded joint position and of the variable laser power, together with the top picture of the joint are shown in Figure 5. The photodiode signal exhibits only a slight decrease of its mean value as far as the laser power is lowered.

**Figure 6.**
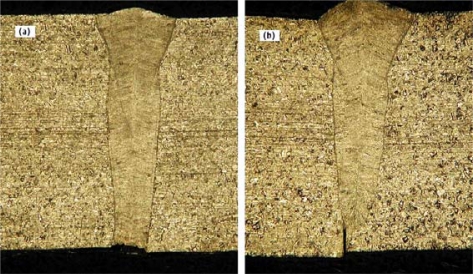
Macrographs of the joint cross sections: (a) full penetration: P = 1.8 kW, v = 80 mm/s, Q_He_ = 100 L/min (zone 1); (b) partial penetration: P = 1.0 kW, v = 80 mm/s, Q_He_ = 100 L/min (zone 3).

A metallographic inspection of the weld cross sections revealed that the decrease of the laser power caused a lack of penetration in the last segment of the joint (Figure 6).

**Figure 7.**
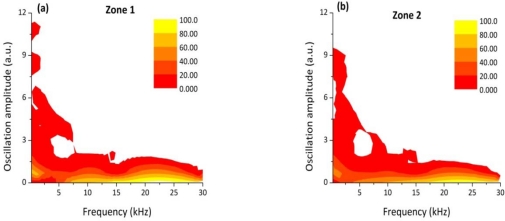

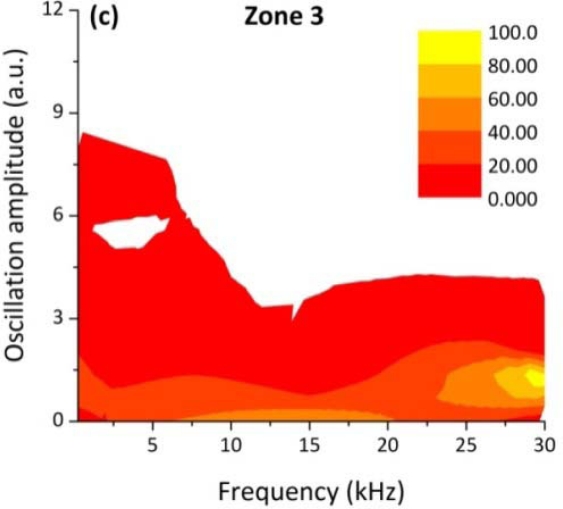
Relative oscillation amplitude of the frequency bands of the photodiode signal acquired during a welding process performed with decreasing laser power (Zone 1: P = [1.8−1.5 kW], Zone 2: P = [1.5−1.2 kW], Zone 3: P = [1.2−0.9 kW]).

The distributions of the relative oscillation frequency intensities for each sector are reported in Figure 7.

It can be noticed that for a fully penetrated keyhole (zone 1), the DWT map reproduces the same behaviour of the frequency distribution observed in case of a defect-free weld: the bands between 200 Hz and 15 kHz are more intense than the bands at higher frequencies. As soon as the laser power was reduced we observed that the amplitude of the oscillations in the frequency bands above 15 kHz progressively became more intense and reached the maximum value in the partially penetrated joint segment (zone 3). Accordingly, the amplitude of the frequency bands below 10 kHz dropped down.

**Figure 8.**
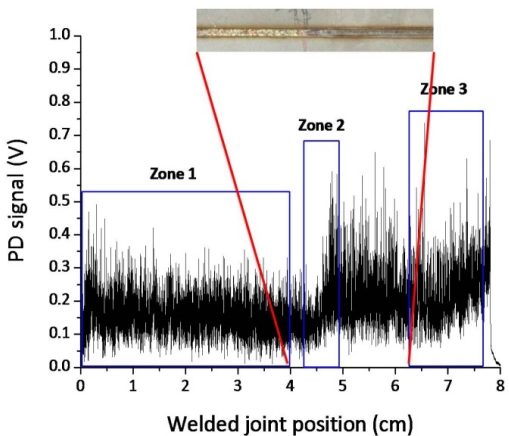
(a) Photodiode signal detected obtained for variable gas flow rate. Welding parameters: P = 1.8 kW, v = 80 mm/s, Q = from 100 L/min (Zone1) to 0 L/min (Zone 3); (b) A particular of the top view of the welded joint in the transition zone between efficient and inefficient shielding conditions.

The experimental results presented in Figure 7 clearly show that a correlation exist between the frequency components of the oscillation of the plasma plume optical emission and the weld penetration depth. The higher frequency modes of oscillation (above 15 kHz) have a stronger contribution when a shallower penetration occurs while, in case of fully penetrated joints, the frequency bands below 15 kHz play a dominant role. These results can be explained by considering the dynamics behavior of the keyhole. Theoretical models [13–17] showed that the keyhole is able to perform radial, axial and azimuthal oscillations. While typical radial frequencies are predicted between 500 and 3,500 Hz, depending on the beam radius, the axial and azimuthal eigenfrequencies, most likely related to the penetration depth were found in the range above several kHz. Finally, we carried out a welding test in which the Helium gas flow rate was progressively reduced during the process, starting from 100 l/min, so that after about 6 cm a completely inefficient gas shielding was established. The corresponding acquired optical signal as a function of the welded joint position together with the top picture of the joint are shown in Figure 8.

**Figure 9.**
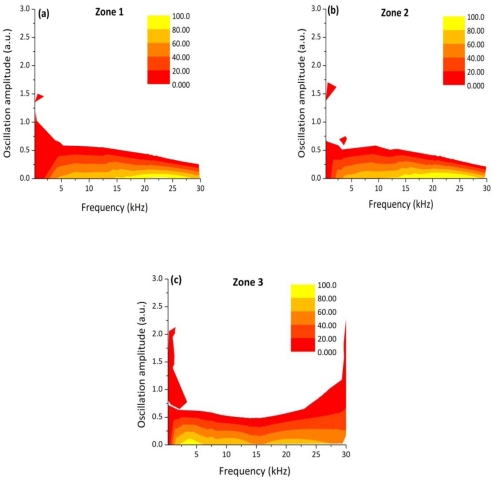
Relative oscillation amplitude of the frequency bands of the photodiode signal acquired during a welding process performed with decreasing laser power (Zone 1: Q = 100 L/min, Zone 2: transition zone, Zone 3: Q = 0 L/min).

The DWT frequency distribution is analyzed in three different sectors, as reported in Figure 9.

For optimal shielding gas conditions (Zone 1) the frequency bands between 200 Hz and 5 kHz are more intense than the bands at higher frequencies, as in the previous cases. As soon as the gas flow rate has been closed, the frequency map shows that the amplitude of the oscillations in the frequency above 15 kHz become very intense, while the oscillations in the range between 500 Hz and 15 kHz show almost the same intensities. From these results it can be argued that the shielding gas which was inflated almost coaxially to the laser beam interferes with the plasma plume ejected by the keyhole surface thus affecting the axial and azimuthal capillary oscillations. Consequently a sudden change of the shielding conditions or a complete lack of shielding not only determines a seam surface oxidation but involves a rapid grow of the signal frequency contribution above 15 kHz.

Table 2 summarizes the relationship between the welding parameters variations, the induced defects and the corresponding DWT behavior.

## 4. Conclusions

In this work we have experimentally demonstrated that by applying the discrete wavelet transform (DWT) tool to the optical emission signals, acquired through a Si-PIN photodiode, during a laser welding process, it is possible to obtain information on the change of process conditions due to variations of incident laser power or shielding gas flow. The experimental investigation was limited to the CO_2_ laser welding process of stainless steel sheets in butt-joint configuration.

The whole acquired signal was divided into several weld segments corresponding to different process conditions. Each signal segment was decomposed into seven frequency bands from 200 Hz to 30 kHz. A distinctive frequency distribution under different welding conditions was found. The decrease of laser power determining a lack of penetration was detected as an increase of the frequency components above 15 kHz. An abrupt change of the same frequency bands was found in case of a change of gas shielding conditions causing surface oxidation of the joint. These results are consistent with previous models predicting the axial and azimuthal oscillations of the keyhole in the same frequency band.

Differently from other signal processing algorithms, the DWT analysis is capable of providing the time and frequency information simultaneously, thus showing a great potential for real time process control applications. Given a signal with a length of N acquired points, the DWT decomposes it into maximum log_2_N components. Therefore if the signal is sampled with a rate of 60kHz, the number of points necessary to analyze frequencies up to 30 kHz is at least N = 150. In this way, for a processing speed of 80 mm/s, the highest achievable spatial resolution on the welded joint was in our case about 0.2 mm. This kind of resolution is compatible with a process control application. The real time capability of the system to react to a change of the process conditions, will obviously depend on the CPU computation rate.

Further laser welding experiments are planned to test the reliability of this technique on different metal alloys and/or joint geometries. Moreover we intend to investigate if the DWT analysis is able to detect other kinds of weld defects.

## Figures and Tables

**Table 1.** Frequency bandwidths.

**Level**	**Bandwidth [kHz]**
1	15–30
2	7.5–15
3	3.75–7.5
4	1.875–3.75
5	0.937–1.875
6	0.468–0.937
7	0.234–0.468

**Table 2.** DWT behavior as a function of the operating parameter and the detected defects (*induced defects have been recognized by metallographic or visual inspections of the welded joints).

**Process parameter variation**	Defect *	**DWT behavior**
Laser power decreasing from 1.8 kW to 0.9 kW	Lack of penetration	Increase of the 15–30 kHz band components
Lack of shielding	Seam oxidation	Increase of the 20–30 kHz band components
